# With respect to coefficient of linear thermal expansion, bacterial vegetative cells and spores resemble plastics and metals, respectively

**DOI:** 10.1186/1477-3155-11-33

**Published:** 2013-10-09

**Authors:** Koichi Nakanishi, Akinori Kogure, Takenao Fujii, Ryohei Kokawa, Keiji Deuchi, Ritsuko Kuwana, Hiromu Takamatsu

**Affiliations:** 1 Research Laboratories for Beverages, Kirin Company, Limited, Technovillage 3F, 1-17-1 Namamugi, Tsurumi-ku, Yokohama 230-8628, Japan; 2 Shimadzu Techno-Research, 380–1 Horiyamashita, Hadano, Kanagawa 259-1304, Japan; 3 Analytical & Measuring Instrument Division, Shimadzu Corporation, 1 Nishinokyo, Kuwabara-cho, Nakagyo-ku, Kyoto 604-8511, Japan; 4 Faculty of Pharmaceutical Sciences, Setsunan University, 45–1 Nagaotoge-cho, Hirakata, Osaka 573-0101, Japan

**Keywords:** Scanning probe microscope, Nano thermal analysis, Coefficient of liner thermal expansion, Transition temperature, Microorganisms, Spore

## Abstract

**Background:**

If a fixed stress is applied to the three-dimensional z-axis of a solid material, followed by heating, the amount of thermal expansion increases according to a fixed coefficient of thermal expansion. When expansion is plotted against temperature, the transition temperature at which the physical properties of the material change is at the apex of the curve. The composition of a microbial cell depends on the species and condition of the cell; consequently, the rate of thermal expansion and the transition temperature also depend on the species and condition of the cell. We have developed a method for measuring the coefficient of thermal expansion and the transition temperature of cells using a nano thermal analysis system in order to study the physical nature of the cells.

**Results:**

The tendency was seen that among vegetative cells, the Gram-negative *Escherichia coli* and *Pseudomonas aeruginosa* have higher coefficients of linear expansion and lower transition temperatures than the Gram-positive *Staphylococcus aureus* and *Bacillus subtilis*. On the other hand, spores, which have low water content, overall showed lower coefficients of linear expansion and higher transition temperatures than vegetative cells. Comparing these trends to non-microbial materials, vegetative cells showed phenomenon similar to plastics and spores showed behaviour similar to metals with regards to the coefficient of liner thermal expansion.

**Conclusions:**

We show that vegetative cells occur phenomenon of similar to plastics and spores to metals with regard to the coefficient of liner thermal expansion. Cells may be characterized by the coefficient of linear expansion as a physical index; the coefficient of linear expansion may also characterize cells structurally since it relates to volumetric changes, surface area changes, the degree of expansion of water contained within the cell, and the intensity of the internal stress on the cellular membrane. The coefficient of linear expansion holds promise as a new index for furthering the understanding of the characteristics of cells. It is likely to be a powerful tool for investigating changes in the rate of expansion and also in understanding the physical properties of cells.

## Background

When solid materials are heated, they expand and generally exhibit a thermal creep curve. If a fixed stress is applied to the three-dimensional z-axis of a material and the material is then heated, the amount of expansion resulting from thermal stress increases according to a fixed coefficient of thermal expansion [1]. As the temperature approaches the transition temperature, at which the physical properties of the material change, the rate of expansion decreases and the material reaches its maximum expansion. If expansion is plotted against temperature, the transition temperature is at the apex of the curve. In the case of a solid, the transition temperature may be determined as the melting point of the solid [1]. Different materials, such as metal and plastic, differ in their melting points and their patterns of thermal expansion, so they provide different curves. Microbial cells are not made of a single solid material, but rather of various constituent materials, and the composition varies according to the species and the condition of the cell. The rate of expansion and the transition temperature determined from the amount of expansion thus differ depending on the species and the condition of the cell, and may therefore offer a new approach for the study of cellular structure. However, currently there are no valid methods for determining the rate of expansion and the transition temperature from the amount of expansion of a single cell, and to date there have been no studies conducted on this topic. To address this, we have developed a method for measuring the coefficient of thermal expansion and the transition temperature of microbial samples using a nano thermal analysis (nano-TA) system.

## Results and discussion

### Change in the coefficient of linear expansion and its relationship to the transition temperature, determined by nano-TA-SPM

**Figure 1:**
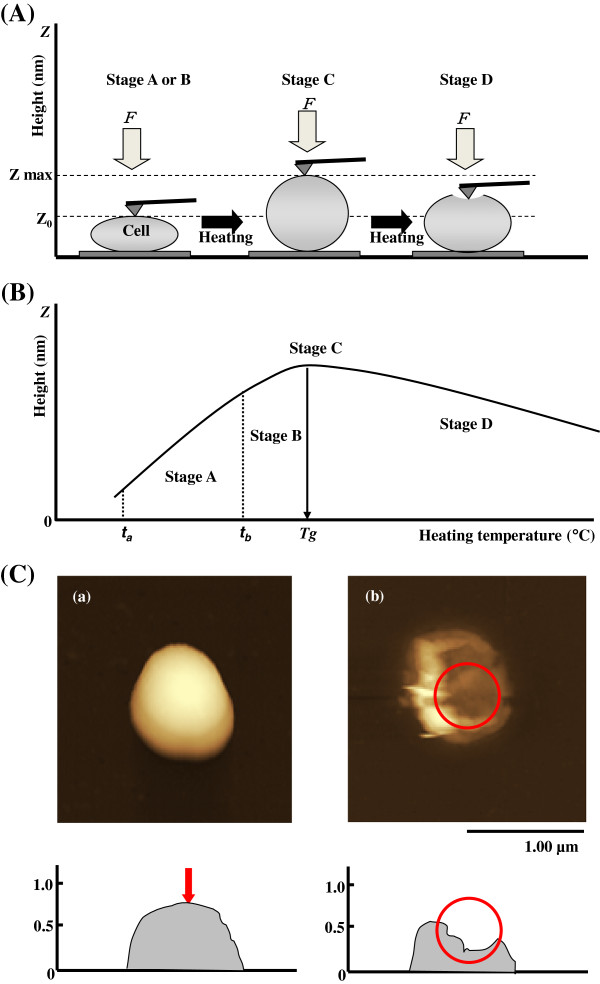
**Principal behind the nano**-**TA**-**SPM system. (A)** Position of cantilever and form change of a cell upon heating. Stage A, the initial heating stage: the coefficient of linear expansion increases, α > 0, and the cell expands at a constant rate. Stage B, the heating stage: the coefficient of linear expansion increases more slowly than stage A, Stage C, the maximum expansion stage: the coefficient of linear expansion has stopped, α = 0, and this temperature is the transition temperature *Tg* of the coefficient of linear expansion [°C]. Stage D, the ruptured stage: the coefficient of linear expansion decreases, α < 0, and the shape of the heated cell or spore is destroyed. Z_0_ is the height at the start of heat application, and z max is the height when the transition temperature is reached. **(B)** The distortion due to expansion after hanging a constant stress from the axial top of Z-value and heating the cell or spore. The change in the physical properties due to heating have occurred when the height = 0 (height Z_0_ in (A)). Stage A is from *t*_*a*_ to *t*_*b*_, where α is constant; stage B is from *t*_*b*_ to *Tg*; stage C is the transition temperature *Tg* of the coefficient of linear expansion; and stage D is where α < 0 and decreases, the cell fuses, and its shape is destroyed. **(C)** Changes in the surface form of spores before and after heating, (a) nano-TA image and cross-sectional diagram of *B. subtilis* before heating, (b) nano-TA image and cross-sectional diagram of *B. subtilis* heated to the transition temperature (*Tg* = 125°C). Red circle shows the change in form as the cell reaches the transition temperature, where expansion due to heating is maximum (coefficient of linear expansion = 0) and the cell partially fuses.

A scanning probe microscope (SPM) combined with a nano-TA system was used to measure the coefficient of linear expansion. This coefficient defines the percentage of thermal expansion from the axial distortion of the z-axis and the transition temperature [2,3]. The cantilever of the SPM was brought to contact the surface of the cell and was applied to force it with a fixed stress. The cell was heated and the amount of expansion monitored, from which the transition temperature was determined. Figure 1 shows the principle behind determining the coefficient of linear expansion, α (Figure 1 (A)), and a model for the change in the amount of distortion of the cell, which gives a creep curve resulting from heating the cell (Figure 1 (B)). Expansion, softening, and distortion of the material accompanying the increase in temperature are measured by changes in the vertical position (changes in height along the z-axis) of the SPM probe in contact with the material. In stage A, the cell expands at a fixed rate due to heating, and the coefficient of linear expansion of the cell can be determined from changes in the z-value. In stage A, the coefficient of linear expansion, α = ∆*L* / *L*_*0*_∆*T*, of the cell is calculated as the rate of increase in the z-value per unit temperature (×10^-6^/°C) [1], where *L*_*0*_ is the height of the microbial cell (nm) along the z-axis before heating, ∆*L* is the amount of expansion of the cell (amount of deflection of the cantilever) (nm) when the temperature of the probe increases from *t*_*a*_ to *t*_*b*_, and ∆*T* is the change in temperature (°C) from *t*_*a*_ to *t*_*b*_. The probe temperature at which the z-value is maximum (z max) is the transition temperature *Tg*. Stage B covers the change in probe temperature from *t*_*b*_ to *Tg*, where the coefficient of linear expansion of the cell decreases to 0. Stage C is at *Tg*, where the coefficient of linear expansion is 0. Stage D is reached if the cell is heated above *Tg*, when the z-value decreases.

### The coefficient of linear expansion and transition temperature of bacteria and yeast

**Figure 2:**
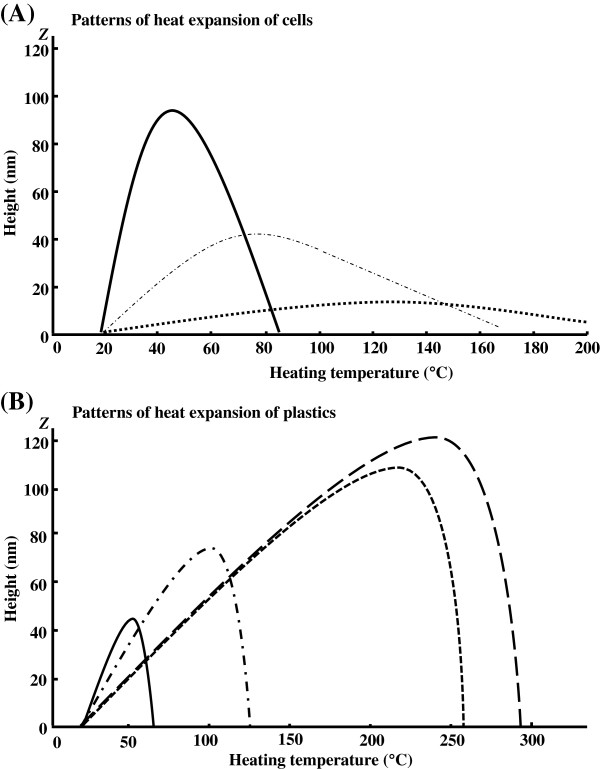
**Changes in the distortion of a cell as a result of expansion due to heating. (A)** Patterns of heat expansion of cells. Continuous line, *E*. *coli* vegetative cells; long dashed dotted line, *B*. *subtilis* vegetative cells; dotted line, *B*. *subtilis* spores. **(B)** Patterns of heat expansion of plastics. Continuous line, PL; long dashed dotted line, PE; dotted line, PET; dashed line: nylon.

The strains investigated were vegetative cells of two strains of Gram-positive bacteria, two strains of Gram-negative bacteria, and one strain of yeast. In addition, spores of five strains of bacteria of the genus *Bacillus* and two strains of anaerobic thermophilic bacteria were also tested. Four types of plastic with different melting points were selected as materials for comparison. The curves showing changes in the amount of expansion resulting from heating the cells and the plastics were compared (Table 1). For the purpose of comparison, the coefficients of linear expansion and the transition temperatures of various metals [4,5] (where the melting point was taken as the transition temperature) are cited in Table 2. Among the vegetative cells, the Gram-negative *Escherichia coli* and *Pseudomonas aeruginosa* have higher coefficients of linear expansion and lower transition temperatures than the Gram-positive *Staphylococcus aureus* and *Bacillus subtilis*. On the other hand, spores, which have low water content [6], overall showed lower coefficients of linear expansion and higher transition temperatures than vegetative cells. Comparing these trends to non-microbial materials, vegetative cells showed behaviour similar to plastics and spores showed behaviour similar to metals with regards to the coefficient of liner thermal expansion. Figure 2 (A) shows changes in the distortion of *E*. *coli* vegetative cells and *B*. *subtilis* spores caused by heat as typical examples of changes in the amount of expansion due to heating. In stage B, *E*. *coli* vegetative cells showed a greater coefficient of linear expansion and z max, but lower *Tg*, than *B*. *subtilis* spores. Also, in stage D, *E*. *coli* vegetative cells showed greater change in z-value than *B*. *subtilis* spores. Figure 2 (B) shows changes in the amount of expansion of four types of plastic. These results show that the degree of deformation of spores as a result of local application of heat is less than that of vegetative cells and plastics (Figure 2).

**Table 1:** The transition temperature and the coefficient of linear expansion of different bacteria, yeast, and plastic materials

**Bacteria, yeast, and plastic materials**	**Transition temperature*Tg*(°C)**	**Coefficient of linear expansion (×10**^**-6**^**/°C)**
Vegetative cells		
*S*. *aureus*	58 ± 0.7	190 ± 10.5
*E*. *coli*	48 ± 0.5	360 ± 13.5
*B*. *subtilis*	71 ± 1.8	105 ± 7.5
*P*. *aeruginosa*	56 ± 0.9	230 ± 16.5
*Saccharomyces pastorianus*	54 ± 1.2	280 ± 12.5
Spores		
*G*. *stearothermophillus*	172 ± 1.2	8 ± 0.3
*B*. *coagulans*	131 ± 2.0	11 ± 0.2
*B*. *subtilis*	125 ± 2.5	14 ± 0.6
*B*. *licheniformis*	107 ± 1.1	19 ± 0.5
*B*. *megaterium*	113 ± 0.9	18 ± 0.5
*T*. *mathranii*	239 ± 6.1	5 ± 0.2
*M*. *thermoacetica*	289 ± 6.7	4 ± 0.3
Plastic materials		
PCL	55	122 ± 3.5
PE	116	102 ± 7.5
PET	235	70 ± 5.5
Nylon	256	65 ± 4.5

Number of measured samples = 20.

*PCL*: Polycaprolactone, *PE*: Polyethylene, *PET*: Polyethylene terephtalate.

**Table 2:** **Coefficient of thermal expansion (coefficient of linear expansion) of metals **[4,5]

**Metals**	**Melting point (°C)**	**Coefficient of linear expansion (×10**^**-6**^**/°C)(20°C)**
Iron	1539	11.7
Copper	1085	16.7
Gold	1064	14.1
Aluminium	660	23.1
Magnesium	650	24.8
Zinc	420	29.7

### Comparison of the coefficient of linear expansion and transition temperature between bacteria, yeast, and materials

**Figure 3:**
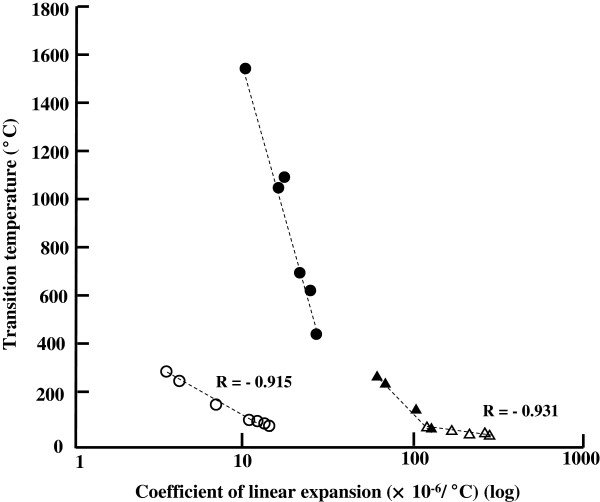
**Comparison of the coefficient of linear expansion of vegetative cells,bacterial spores,plastics and metals.** Open circles, bacterial spores; open triangles, vegetative cells; closed circles, metals; closed triangles, plastics.

Figure 1 (C) shows SPM images before and after heating of the deformation of a spore in contact with the probe. The transition temperature is the temperature at which some physical properties of the microbial cell changes due to heating, it is like the melting point which changes state of the material. This is the thermal death temperature, at which the structure of the microorganism undergoes irreversible physical damage. Figure 3 shows the transition temperature plotted against the coefficient of linear expansion for the vegetative cells and spores and in Table 1, it can be seen that there is a strong negative correlation between transition temperature and coefficient of linear expansion for both vegetative cells (open triangles in Figure 3, r = -0.931) and spores (open circles in Figure 3, r = -0.915). Figure 3 displays each coefficient of linear expansion and transition temperature (or melting point) of vegetative cells, bacterial spores, plastics and metals on one graph. Although the transition temperatures of plastics and metals differ from that of vegetative cells and bacterial spores, the coefficient of linear expansion of spores is similar to that of metals. Cells may be characterized by the coefficient of linear expansion as a physical index, and the coefficient of linear expansion may also characterize cells structurally since it relates to volumetric changes, surface area changes, the degree of expansion of water contained within the cell, and the intensity of the internal stress on the cellular membrane. The coefficient of linear expansion holds promise as a new index for furthering the understanding of the characteristics of cells. It is likely to be a powerful tool for investigating changes in the rate of expansion and also in understanding the physical properties of cells.

## Conclusions

Cells may be characterized by the coefficient of linear expansion as a physical index, and the coefficient of linear expansion may also characterize cells structurally since it relates to volumetric changes, surface area changes, the degree of expansion of water contained within the cell, and the intensity of the internal stress on the cellular membrane. The coefficient of linear expansion holds promise as a new index for furthering the understanding of the characteristics of cells. One of the problems in the future includes a difference in the water content of the vegetative cells and the spores. Estimating the possibility to influence the difference that this difference is a coefficient of linear thermal expansion to be high. We would investigate whether the quantity of water contained in a cell effects on the coefficient of linear thermal expansion. It is likely to be a powerful tool for investigating changes in the rate of expansion and also in understanding the physical properties of cells.

## Methods

### Bacterial and yeast strains

The spores used were prepared from *Geobacillus stearothermophilus* NBRC 13737, *Bacillus coagulans* DSM 1, *B*. *subtilis* NBRC 13719T, *B*. *megaterium* NBRC 15308T, *B*. *licheniformis* NBRC 12200, *Thermoanaerobacter mathranii* DSM 11426, and *Moorella thermoacetica* DSM521T. The vegetative cells were *Staphylococcus aureus* NBRC 100910, *Escherichia coli* IFO 3301, *B*. *subtilis* NBRC 13719T, and *Pseudomonas aeruginosa* ATCC 10145, and the yeast *Saccharomyces pastorianus* RIB 2010.

### Culture and pretreatment methods

Bacteria were cultured in nutrient broth (Difco, Becton Dickinson and Co., Franklin Lakes, NJ, USA); *G*. *stearothermophilus* was cultured at 60°C; all other bacteria were cultured at 35°C. For vegetative cells, the log phase (OD_600_ = 0.8 – 1.0) after 4 to 12 h of culture was used. Where spores were used, the bacteria were cultured under the same conditions for 96 h [7]. For *T*. *mathranii* and *M*. *thermoacetica* spores, the bacteria were cultured in modified TGC culture medium (Nissui Pharmaceutical Co., Ltd., Tokyo, Japan) at 60°C for 72 h. The yeast *S*. *pastorianus* was cultured in YM Broth (Difco, Becton Dickinson) at 25°C for 48 h. Spores were collected from the culture fluid as reported previously [7].

### Plastic materials

The thin films of the plastic materials used were polycaprolactone (PCL; Tm = 55°C, Wako, Tokyo, Japan) and polyethylene (PE; Tm = 116°C, Wako, Tokyo, Japan), which were processed into thin films, as well as polyethylene terephtalate (PET; Tm = 235°C, Pana Chemical Co., Ltd., Tokyo, Japan) and polyamide 66 (nylon 66; Tm = 256°C, Murakami Dengyo Co., Ltd., Yokohama, Japan).

### Measurement of transition temperature and coefficient of linear thermal expansion of bacteria

A Nano Search Microscope type SFT-3500 (Shimadzu Corporation, Kyoto, Japan) was combined with a nano-TA system (nano thermal analysis) (Anasys Instruments, Santa Barbara, CA, USA) [2,3]. The cantilever was brought into contact with a single microbial cell at a constant stress of 200 nN and heated from 25°C at 10°C/s to a temperature of 100°C or 400°C, continuously. The measurement point was the highest point, determined as reported previously [7].

## Competing interests

The authors declare that they have no competing interests.

## Authors’ contributions

KN designed the experiments, analysed data, and prepared the manuscript. AK, TF and RK developed and supervised the use of the Nano Search microscope SFT-3500 for microbiological research. RK and HT lead the effort to clarify the relationship between the coefficient of linear expansion and transition temperature of a microbial cell, and the heat-resistance relationship. KD supervised the experiments. All authors discussed the results and approved the final manuscript.
